# Aneurism on the Common Carotid Artery; Deligation of the Vessel; Death

**Published:** 1851-08

**Authors:** 


*Aneurism on the Common Carotid Artery; Deligation of the Vessel; Death.*—Our readers may remember that the carotid artery was tied some months ago, with perfect success, by Mr. Johnston, at St. George's Hospital. The delegation of the vessel was here undertaken for the sake of commanding hæmorrhage from the interior of the mouth, brought on by the thrust therein of the point of a parasol. The boy did extremely well, and presented none of the cerebral symptoms which are said sometimes to follow the interrupted supply of the blood to the brain.

We have now to put upon record an operation of the same kind, performed upon an adult, for the cure of an aneurism of the carotid artery. Though many methods have by turns been proposed to procure the obliteration of the sac,—as pressure, injection, and galvano-puncture, the results have been so unsatisfactory, that the only means likely to afford permanent relief is the deligation of the vessel on the cardiac side of the tumor. Such deligation seems, however, somewhat hazardous, when so important a trunk as the carotid artery is to be tied; but the cerebral disturbance which might naturally be looked forward to, is in fact very seldom noticed.

It is well known that we are indebted to chance for the discovery of the inocuity of this operation; a surgeon wounds the carotid whilst removing a scirrhous tumor from the side of the face, and to save his patient's life, he ties the vessel, and the case does well. Abernethy was the first, in this country, who attempted the operation; Fleming followed his example, and the deligation of the carotid artery soon became an established and acknowledged operation in surgery. The vessel has been several times tied for the cure of aueurism by anastomosis, or erectile tumors in the cheek or eye: Mr. Travers, Mr. Dalrymple, Mr. Wardrop, have successfully operated in this country in cases of this nature.

When the deligation is undertaken for the cure of aneurism, it is of course very advantageous that the tumor should be so situated as to allow of the ligature being applied in that portion of the course of the artery which offers the greatest facility for such operation—viz. in the upper triangle of the neck. But aneurisimal tumors are sometimes situated so low down, as to leave but very little room between their lower border and the clavicle; here the dissection will of course be longer, and the whole operation require much caution. Dr. Robertson, of Edinburgh, operated in a case of carotid aneurism where the sac was situated low down, and the tumor had burst into the œsophagus; he made his incision to the internal side of the sterno-mastoid muscle only one inch in length, and succeeded in securing the vessel. Mr. Fergusson had to surmount a difficulty of the same kind, as the tumor came close to the clavicle ; the artery was, however, laid bare and tied, after a careful dissection, which presented more than usual difficulty.

Mary S——, aged thirty-eight, and unmarried, was admitted into King's College Hospital, June 11, 1851, under the care of Mr. Fergusson, with a swelling on the left side of the neck, presenting several symptoms of carotid aneurism. The patient has generally enjoyed good health, but has led a dissipated life, and endured great privations. None of her family have suffered from diseased arteries or aneurism. About eighteen months ago she was under treatment at the Westminister Hospital for gangrene of the toes, induced by exposure to wet and cold during two days and nights. Six months since, the patient began to cough, and to experience difficulty of swallowing ; about the same time she likewise noticed a small swelling in the course of the carotid artery on the left side, which, however, gave her no pain. The swelling gradually increased to its present size, and the function of respiration became more and more impeded.

On examination, a tumor, about the size of a small fist, tense, pulsating, and tender to pressure, was noticed on the left side of the neck, over the course of the carotid artery. It extended from the base of the jaw nearly to the upper border of the clavicle. No morbid sound of the heart could be detected, but a distinct bruit was heard in the tumor ; the general arterial system seemed healthy. The breathing was very difficult. On the third day after admission, the patient became very low and weakly ; the dyspnœa was great, as the tumor seemed somewhat against the trachea and larynx. The skin over the swelling was becoming very red and tense.

Mr. Fergusson examined the patient just before proceeding with several other operations ; and finding her with so much difficulty of breathing, and the tumor threatening to burst, he determined to tie the carotid artery on the cardiac side of the tumor. The patient had become suddenly worse, and this circumstance necessitated prompt measures; but it had been Mr. Fergusson's intention to use appropriate internal remedies before undertaking the operation—a precaution which would certainly in many instances be extremely useful.

No delay could, however, intervene in this instance, for there was every probability of the tumor bursting outwardly, as it presented a highly inflamed spot, or the patient dying from suffocation. When she was brought into the theatre, the question arose whether, in her weak state, the patient should inhale chloroform. Mr. Fergusson thought that the anæsthetic agent should be administered, but with great caution.

When the patient was completely insensible, or nearly so, and the head slightly raised, Mr. Fergusson commenced with a longitudinal incision, about three inches long, to the inner side of the mastoid muscle, from the lower portion of the tumor down along the upper end of the sternum. After the cellular tissue, fascia, and platysma had been divided, the sterno-mastoid came into view, and as here the vessel lay very deep, Mr. Fergusson cut this muscle across. The sterno-hyoid and thyroid were now carefully divided, as well as some loose cellular tissue, and the sheath of the vessels was perceived. This was cautiously opened upon the director, and the aneurism-needle passed round the carotid artery from without inwards, when the vessel was well secured by a strong thread. Temporary tightening caused the pulsations of the tumor to cease, and when this had been ascertained, the ligature was finally fixed, and the lips of the wound brought together by suture.

No cerebral symptom became manifest after the deligation of the artery, but the pulsations of the radials became much slower; the breathing, which had been very labored, regained some vigor, and the patient was removed in a comparatively satisfactory state.

Mr. Fergusson took occasion to remark, that he had not expected when the patient was admitted into the Hospital, to operate so soon, as he intended to afford her eight or ten days' rest and appropriate treatment; but the disease had progressed so rapidly, inflammation had set in so quickly, and the dyspnœa was so great, that if not interfered with, the tumor would have burst either internally or externally. On consultation with his colleagues he had expressed his conviction, and they had agreed, that the vessel should be tied forthwith, though the space between the clavicle and the lower portion of the tumor was very short. The aneurism was probably situated on the upper portion of the common carotid, or just at the bifurcation, but to know the exact position was not important as regarded treatment.

He (Mr. Fergusson) had made his incision very long, reaching even over the sternum, as the vessels lie so deep in this locality that plenty of room is very necessary, and on this ground he had divided the sterno-mastoid muscle without hesitation, the more so as this operation is one involving life, and that a muscle was of little importance in comparison. Mr. Fergusson had been somewhat apprehensive of the small veins in that region, in direct communication with the veins of the neck, but the hæmorrhage had altogether been trifling; the jugular vein was not seen at all. The best and only step had now been taken for the patient's safety; she might, however, now die from the bursting of the sac either externally or internally. A favorable circumstance in the operation was, that the patient had a very long neck, and was very thin.

At four o'clock in the afternoon the effects of chloroform had been recovered from, the breathing was more regular than previous to the operation, but there was an accumulation of mucus in the air passages which the patient could not force away. She was given squill and hyoscyamus in camphor mixture. At seven in the evening respiration was still less embarrassed, the pulse 90, the tumor slightly diminished in size, and very tense; but towards three o'clock in the morning the patient was seized with a short convulsive fit, and died soon afterwards, without any previous impairment of cerebral influence.

On a post-mortem examination the parietes of the heart were found very thin and weak; the arterial system immediately above that organ did not exhibit any striking alteration, except the abdominal aorta, where abundant atheromatous deposit was found; the other organs of the chest were healthy. The ligature was firmly fixed, and situated about an inch and a half below the tumor; the latter was filled with coagulum and soft fibrinous masses, and was situated opposite the thyroid body, extending as high as the os hyoides. The aneurism was of the description in which all the coats of the vessels had originally been equally distended; the opening of the artery into the lower part of the sac was smooth and round, and continuous with the walls of the tumor; the upper aperture was about of the same kind, having a slight valve added to it, and the carotid artery, at its emergence, was only half the calibre of the portion which was seen entering the sac. The whole of the latter lay on the left lobe of the thyroid body, on the trachea and œsophagus, these organs being evidently compressed by the tumor.

The vagus nerve, imbedded in the walls of the sac, was seen entering and emerging from these walls, close to the artery; in gently pulling the nerve above and below, its outline could be seen tightening along the parietes of the sac. This peculiar disposition of the vagus may be looked upon as greatly conducive to the intense dyspnœa which the patient suffered for the last week of her life.

The diagnosis of this case did not offer difficulties of a serious nature, but it will be conceded that too much care cannot be bestowed upon the recognition of the actual nature of the disease; for it is principally in the neck that tumors and abscess may simulate aneurism. Enlarged glands are especially liable to create deception; but it is seldom that the thrill communicated to the hand, or the bruit conveyed to the ear, are so distinct as in true aneurism, as was observable in the foregoing case. The full dilatation of all the coats of the vessels took place in a comparatively short time, six months having only elapsed since the patient perceived the swelling; for such cases as the one related by Mr. Hodgson, where a femoral aneurism destroyed an old man in three weeks, are certainly very rare. It will be perceived that Mr. Fergusson's patient was a female; we need not point out how seldom aneurisms affect women, this fact lending, as we venture to think, much weight to the supposition that violent exertion has much influence on the formation of aneurismal tumors, where vessels are not in a sound state.—*London Lancet.*

